# Toll-Like Receptor 4 Engagement Drives Differentiation of Human and Murine Dendritic Cells from a Pro- into an Anti-Inflammatory Mode

**DOI:** 10.1371/journal.pone.0054879

**Published:** 2013-02-11

**Authors:** Romana Luger, Sneha Valookaran, Natalie Knapp, Caterina Vizzardelli, Alexander M. Dohnal, Thomas Felzmann

**Affiliations:** 1 St. Anna Children’s Cancer Research Institute, Laboratory of Tumor Immunology, Department of Pediatrics, Medical University Vienna, Austria; 2 Activartis Biotech GmbH, Vienna, Austria; University Medical Center Freiburg, Germany

## Abstract

The dendritic cell (DC) coordinates innate and adaptive immunity to fight infections and cancer. Our observations reveal that DCs exposed to the microbial danger signal lipopolysaccharide (LPS) in the presence of interferon-γ (IFN-γ) acquire a continuously changing activation/maturation phenotype. The DCs’ initial mode of action is pro-inflammatory via up-regulation among others of the signaling molecule interleukin (IL) 12, which polarizes IFN-γ secreting type 1 helper T-cells (Th1). Within 24 hours the same DC switches from the pro- into an anti-inflammatory phenotype. This is mediated by autocrine IL-10 release and secretion of soluble IL-2 receptor alpha (sIL-2RA) molecules. T-cells, when contacted with DCs during their anti-inflammatory phase loose their proliferative capacity and develop regulatory T-cell (Treg) -like anti-inflammatory functions indicated by IL-10 secretion and elevated FoxP3 levels. Studying the kinetics of IL-12 and IL-10 expression from LPS/IFN-γ activated myeloid DCs on a single cell level confirmed these observations. When T-cells are separated from DCs within 24 hours, they are spared from the anti-inflammatory DC activity. We conclude that, in addition to differentiation of DCs into distinct subsets, the observed sequential functional phases of DC differentiation permit the fine-tuning of an immune response. A better understanding of time-kinetic DC features is required for optimally exploiting the therapeutic capacity of DCs in cancer immune therapy.

## Introduction

During the last decades, dendritic cells (DCs) have been identified as the most important regulatory elements in orchestrating immune responses [1]. Studies using primary mouse DCs collected from lymphoid organs, skin and other tissues, suggest that immunity is directed by DC subsets, each of which separately executes a distinct function [2]. Confirmation of such DC subset-mediated immune regulation in humans is complicated by the fact that primary human tissue DCs are not directly available. The majority of information regarding human DCs is derived from DCs differentiated in vitro from monocytes [3], [4], [5]. Such studies have revealed an interesting phenomenon: rather than a direct differentiation into DC subtypes, time dependent changes of the DCs’ function were observed. This pattern of DC differentiation might represent an additional layer of immune regulation.

DCs respond to the notion of danger [6] that comes in different guises to initiate an activation or differentiation process, conventionally referred to as maturation. Maturation results from contact with pathogen- [7] or damage-associated [8] molecular patterns, from contact with pro-inflammatory cytokines [9], or through CD40/CD40L interaction [10], [11], [12], [13]. Binding of microbial pattern molecules such as lipopolysaccharides (LPS) to Toll-like receptors (TLR) on DCs signal danger. Soon after TLR engagement, DCs assume a potent immune stimulatory phenotype characterized by the release of IL-12 for approximately one day [14]. IL-12 secreting DCs trigger robust type 1 T-helper (Th1) cell and cytotoxic T-lymphocyte (CTL) dominated immune responses in vitro [4], [15] as well as in vivo [16], [17].

TLR engagement, however, induces not only pro-inflammatory IL-12 but also anti-inflammatory IL-10 secretion from DCs. IL-10 plays a key role as feedback regulator in Th2 and Th17 cells [18] and in regulatory T-cell (Treg) mediated immune suppressive functions [19]. In addition to IL-10, other molecules known to contribute to immune suppression become active: secretion of soluble IL-2 receptor alpha molecules (sIL2RA, sCD25) [20], phosphorylation of STAT3 [21], up-regulation of indoleamine-2,3-dioxygenase (IDO) [22]. IDO renders activated T-cells susceptible to apoptosis and contributes to Treg activation [23]. Moreover, DCs are linked by their expression of the IL-12 family members IL-27 and IL-23 to immune-regulation and to the maintenance of Th17 cells [24].

These observations hint at a DC differentiation program that initially polarizes pro-inflammatory Th1-dominated immune responses. Approximately 1 day after exposure to a maturation agent, the DCs switch into an anti-inflammatory immune regulatory mode of action. Limiting LPS/IFN-γ-mediated DC maturation to 6 hours enables the priming of T-cells in vitro [15] or in vivo [17] while IL-12 is still released from DCs. This strategy is employed in a clinical development program for cancer immune therapy with tumor antigen charged autologous DCs [25]. As in model systems, patients’ DCs were exposed to LPS/IFN-γ for only 6 hours and could, therefore, be applied to cancer patients in their pro-inflammatory mode of action characterized by IL-12 secretion.

The DC maturation program continues despite withdrawal of LPS/IFN-γ at the time the DCs are transferred into a co-culture with T-cells, inoculated into test animals, or used to treat cancer patients. Eventually DCs switch into their anti-inflammatory phenotype [5]. Thus, it seems reasonable to suppose that T-cells primed by contact with DCs in their pro-inflammatory mode of action would subsequently receive negative-regulatory signals from DCs that continued their differentiation into the anti-inflammatory mode, if the DC/T-cell contact is maintained. In this study, we demonstrate that the separation of DCs from T-cells after a few hours of co-cultivation improves the immune stimulatory effect on T-cells by not only allowing DC/T-cell interaction in the presence of IL-12 release but also by preventing the DCs from delivering their negative immune regulatory signals during their late anti-inflammatory mode. A DC/T-cell contact limited to 6 hours results in significantly improved T-cell activation dominated by Th1 immune polarization as well as reduced Treg activity compared to a continuous DC/T-cell co-culture. Single cell analysis of DCs revealed that the entire differentiation program occurs within one and the same DC. Thus, in addition to the distinct DC subset control of immune responses, we observe time-dependent changes in DC functions from a pro- into an anti-inflammatory mode of action.

## Results

### Characterization of LPS/IFN-γ Matured Myeloid DCs

**Figure 1:**
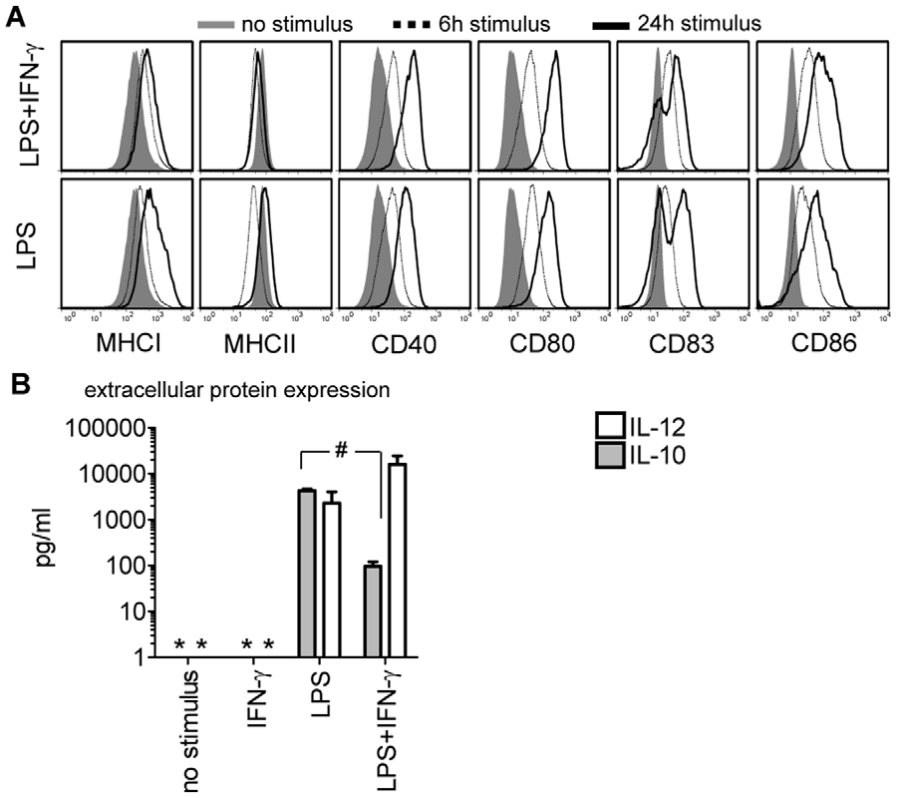
Detection of immune-modifying molecules in maturing human DCs. (A) DC membrane molecule expression at 6 (dotted line) and 24 hours (black line) after LPS or LPS/IFN-γ exposure compared to un-stimulated human DCs (filled gray histograms). One representative out of 3 experiments is shown (donor C). (B) LPS or LPS/IFN-γ induced IL-12 (white bars) or IL-10 (grey bars) protein secretion was measured 48 hours after exposure to the different stimuli (p = 0.006). As a control, DCs were stimulated with IFN-γ or cultured without stimulation for 48 hours. Data are pooled from 4 experiments and depicted as median±SEM (donors A–D). Asterisks indicate cytokine levels below the detection limit of the assay.

Exposure of immature DCs to maturation agents triggers up-regulated expression density of characteristic DC membrane molecules in humans and mice. At 24 hours after initiation of maturation with LPS or LPS/IFN-γ we confirmed enhanced expression of the DC marker molecule CD83, the co-stimulatory molecules CD80 and CD86, the maturation molecule CD40, and of MHC I and II molecules in human monocyte derived DCs. The induction of these molecules became evident 6 hours after exposure to the maturation agents (figure 1A) and was found on bone-marrow derived murine DCs as well (BM-DCs, data not shown). Generally, addition of IFN-γ to the LPS stimulus caused more pronounced CD80, CD86 and MHC I/II expression, and enhanced CD83 expression. DCs, stimulated with a mediator cocktail comprised of tumor necrosis factor alpha (TNF-α), prostaglandin (PG) -E2, IL-1β, and IL-6 exhibited a membrane molecule expression pattern similar to LPS or LPS/IFN-γ exposed DCs (data not shown).

DCs release molecules with opposing functions like IL-12 and IL-10 [14]. IL-12 is secreted from activated DCs, starting during the first 6 hours after LPS/IFN-γ stimulation (table 1). At the same time IL-1β (9.5±2.2 ng/ml) and TNF-α (8.2±1.5 ng/ml) were detected (data not shown). IL-10 secretion started after the peak expression of IL-12 at 6 hours and reached its maximum at 12 hours after the stimulation (table 1). While exposure of DCs to LPS alone triggered the release of equal amounts of IL-10 and IL-12, adding IFN-γ to the LPS maturation stimulus slightly increased the secretion of IL-12, it significantly reduced the release of IL-10 (figure 1B). IFN-γ alone was not capable to trigger IL-10 and IL-12 secretion from DCs. No IL-12 secretion was detected in DCs exposed to the mediator cocktail (data not shown).

**Table 1:** sequential expression of freshly secreted IL-12, IL-10 and sIL-2RA of LPS/IFN-γ matured DCs.

	Interval after stimulus (hours)	IL-12 (pg/ml)	IL-10 (pg/ml)	sIL-2RA (ng/ml)
Donor** F	0–6	27150	0*	0*
	6–12	1265	92	0*
	12–24	390	20	3
	24–48	0*	0*	5
Donor E	0–6	363	0*	n.a.
	6–12	232	0*	n.a.
	12–24	42	0*	n.a.
	24–48	0*	1156	n.a.
Donor C	0–12	642	0*	0*
	12–24	61	1997	2
	24–36	130	0*	2

* Below detection limit; n.a., not analyzed.

** Healthy volunteers donating peripheral blood collected by use of leukocyte aphaeresis.

In monocytes and macrophages it is known that phosphorylation of the transcription factor STAT3 leads to down-modulation of immune responses [21]. For this reason we examined the time kinetic of STAT3 phosphorylation in DCs following the encounter of a danger signal using intra-cellular staining and flow cytometric analysis. Only stimulation with LPS alone, which also resulted in enhanced IL-10 release, was capable of inducing STAT3 phosphorylation (figure S1A). Mirroring the kinetic of IL-10 secretion, STAT3 was phosphorylated in 12% of DCs at 12 hours and reached its maximum with STAT3 phosphorylation in 55% of DCs at 24 hours after encountering LPS (figure S1B). No phosphorylation was detected after exposing DCs to LPS/IFN-γ or IFN-γ alone.

### Short DC/T-cell Contact Leads to Enhanced T-cell Proliferation Using LPS/IFN-γ Matured Myeloid DCs

**Figure 2:**
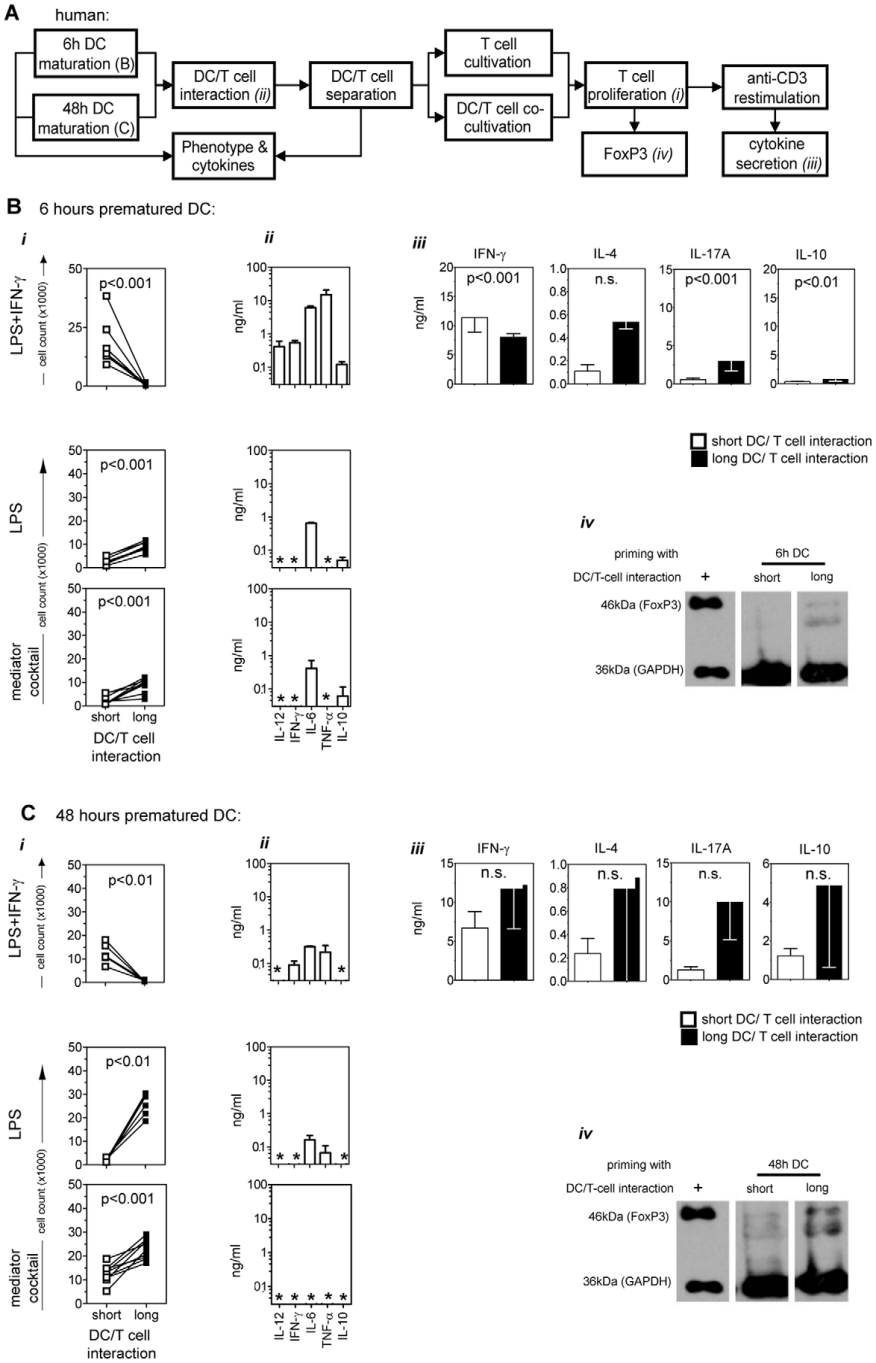
Proliferative capacity and polarization profile in short- versus long-term DC/T-cell co-cultures. (A) Human DCs were matured for 6 or 48 hours, contacted with naïve allogeneic CD4^+^ enriched T-cells for 6 hours, separated using flow sorting, and either maintained alone in culture or re-cultivated with DCs (further-on referred to as “short”-term or “long”-term DC/T-cell interaction, respectively), both for 6 days. After the primary short- or long-term DC/T-cell co-cultures, T-cells were analyzed for FoxP3 expression or, adjusted to equal cell numbers, were re-stimulated with anti-CD3 for 24 hours. The supernatant of the re-stimulated culture was further analyzed for polarization-specific cytokine release. DCs matured for 6 (B) or 48 hours (C) with LPS, LPS/IFN-γ, or mediator cocktail (TNF-α/PG-E2/IL-1β/IL-6) were put in short-term (white squares) or long-term (black squares) co-culture with CD4^+^ T-cells. *(i)* Absolute numbers of proliferating CD4^+^CFSE^−^T-cells after 6 days of DC/T-cell co-culture are given. Pooled data from 8 to 10 experiments are shown. *(ii)* The cytokine composition in the supernatant of the 6 hours T-cell priming co-cultures with 6 or 48 hours matured DCs before sorting are shown. Asterisks indicate cytokine levels below the detection limit of the assay. Pooled data from 3 to 5 experiments are shown as median±SEM. Asterisks indicate cytokine levels below the detection limit of the assay. *(iii)* The culture supernatants of anti-CD3 re-stimulated CD4^+^ T-cells from short- (white squares) or long-term (black squares) primary DC/T-cell co-cultures with LPS/IFN-γ matured DCs (either 6 or 48 hours matured) were analyzed for their content of IFN-γ, IL-4, IL-17A, and IL-10. Three to 10 independent experiments are shown. *(iv)* Detection of FoxP3 in T-cells after short- or long-term DC/T-cell co-culture with LPS/IFN-γ stimulated DCs (matured for 6 or 48 hours). As a positive control (+) the cell lysate of a FoxP3 transfected cell line was used. One representative out of 3 experiments is shown. All data is given as median±SEM. DCs from Donors C, E and F were used in experiments (A) to (C).

The sequential secretion of IL-12 and IL-10 suggested that limiting the priming of T-cells by DCs to the time when IL-12 is predominantly secreted and before IL-10 starts accumulating, favors Th1 polarization and CTL priming. A brief exposure to LPS with and without IFN-γ was used to trigger maturation in human (6 hours) and murine (4 hours) DCs, after which DC/T-cell co-cultures were initiated. All human DC/T-cell co-cultures were discontinued after 6 hours in order to separate T-cells from DCs using flow sorting. Murine T-cells were more sensitive to purification and flow sorting procedures in preliminary experiments, which lead us to prolong murine DC/T-cell co-cultures up to 12 hours before flow sorting T-cells from DCs. This extended contact did not change the net-outcome of the experiments (data not shown). The purified T-cells were then either re-cultivated alone (here termed “short-term co-culture”) or together with the sorted DCs (here termed “long-term co-culture”, figure 2); 6 days in the human system or 3 days in the murine system.

Short-term co-cultures of enriched CD4^+^ T-cells with DCs exposed to LPS/IFN-γ (figure 2B *i)* were significantly more potent in proliferation as compared to long-term co-cultures (p = 0.0001, table 2). This effect was observed using DCs matured for 6 hours (figure 2B *i)* as well as for 48 hours (figure 2C *i)*. As control to our standard LPS/IFN-γ maturation stimulus [25] we used LPS alone or maturation with the pro-inflammatory mediator cocktail [9]. The opposite was observed using DCs matured with LPS alone or with the mediator cocktail: such DCs induced stronger T-cell proliferation in long-term DC/T-cell co-cultures as compared to short-term co-cultures (figure 2B *i*, lower two graph sets).

**Table 2:** Cytokine content of the DC/T-cell co-culture supernatant (6 and 48 hours DC stimulation) before sorting (median±SEM).

	DC	IL-12		IFN-γ		TNF-α		IL-6		IL-10	
	maturation	ng/ml	n	ng/ml	n	ng/ml	n	ng/ml	n	ng/ml	n
LPS	6 h	0*	3	0*	3	0*	2	0.8±0.1	3	0.2±0.1	3
	48 h	0*	3	0*	3	0.1±0	3	0.2±0.1	3	0*	3
LPS/IFN-γ	6 h	0.4±0.2	8	0.4±0.1	8	15.0±6	8	4.2±1.1	8	0.1±0	8
	48 h	0*	5	0.1±0	5	0.2±0.1	5	1.0±0.7	5	0*	5
Mediator cocktail	6 h	0*	3	0*	3	0*	3	0.4±0.3	3	0*	3
	48 h	0*	3	0*	3	0*	3	0*	3	0*	3

* Below the detection limit of the assay.

In preliminary experiments we had observed that the DC/T-cell culture supernatants were essential for re-cultivating the T-cells after flow sorting. By replacing the supernatant with fresh culture medium, the T-cell proliferation was considerably impaired in short-term co-cultures with 6 hours LPS/IFN-γ matured DCs (data not shown). This correlated with enhanced levels of the inflammatory cytokines IL-12, IFN-γ, IL-6 and TNF-α in the co-culture supernatants when LPS/IFN-γ was used as DC stimulus (figure 2B *ii* & C*ii*, table 2). In agreement with the secretion kinetic release of IL-10 was detectable within these DC/T-cell priming cultures.

### Shortened Interaction with DCs Induces IFN-γ in CD4^+^ T-cells While FoxP3 and IL-10 are Decreased

Having observed enhanced CD4^+^ T-cell proliferation by shortening the interaction with LPS/IFN-γ stimulated DCs in humans and mice prompted us to assess the impact of the duration of DC/T-cell contact on immune polarization. The strongest Th1 polarization profile, as indicated by IFN-γ secretion and low amounts of IL-4, IL-17A and IL-10, was induced in T-cells by short interaction with 6 hours stimulated DCs (figure 2B *iii).* During the DC/T-cell co-culture, strong IL-12 release from DCs was detected (figure 2B *ii)*. Although human T-cells, primed in a long-term co-culture with 48 hours matured DCs, released considerable amounts of IFN-γ, they also secreted IL-4, IL-17A and IL-10. Other maturation agents, like LPS alone or the mediator cocktail, induced increased IL-17A and IL-10 levels combined with low IFN-γ levels (data not shown). Short-term co-culture of human CD4^+^ T-cells with 6 hours LPS/IFN-γ stimulated DCs exhibited the strongest Th1 polarization profile and did not promote FoxP3 expression (figure 2B *iv*). The highest induction of FoxP3 was detected in long-term co-cultures using 48 hours matured DCs (figure 2C *iv*).

### Priming of T-cell Receptor Transgenic OT-I/II T-cells by Short and Long Exposure to DCs

**Figure 3:**
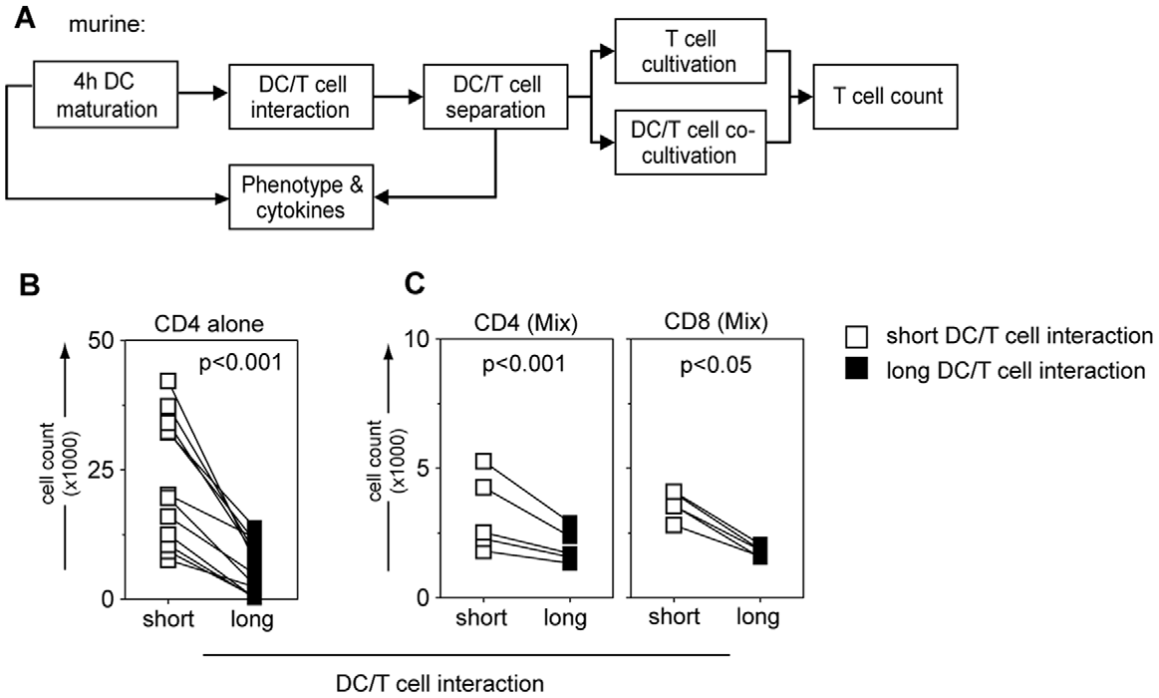
Proliferative capacity and polarization profile in short- versus long-term DC/T-cell co-cultures using murine BM-DC and T-cell receptor transgenic OT-I/II T-cells. (A) Murine BM-DCs were matured for 4 hours, contacted with naïve syngeneic CD4^+^ enriched OT-II T-cells for 12 hours, separated using flow sorting, and either maintained alone in culture or re-cultivated with BM-DCs (further-on referred to as “short”-term or “long”-term DC/T-cell interaction, respectively), both for 3 days. (B) Total cell count of murine enriched CD4^+^ OT-II T-cells after 3 days DC/T-cell co-culture under short-term (white squares) or long-term (black squares) conditions with 4 hours LPS/IFN-γ matured BM-DCs is shown. Pooled data from 12 experiments are shown as median±SEM. (C) A mixture of CD8^+^OT-I and CD4^+^OT-II T-cells was used (OT-I:OT-II = 1∶4) for short-term (white squares) or long-term (black squares) DC/T-cell co-culture, but analyzed on day 3 separately using specific staining for CD4^+^ or CD8^+^ molecules (middle and right graphs, respectively). Pooled data from 5 experiments is shown.

The same proliferation pattern as in the human test system was observed in murine DC/T-cell co-cultures (figure 3B). The cell number of OT-II CD4^+^ T-cells following short-term co-cultivation was significantly higher than following long-term co-cultivation (p = 0.0002; table 3). Next we aimed at assessing the effect of CD4^+^ helper T-cell priming on OT-I CD8^+^ cytolytic T-cells in a 1∶4 mixture in short-term co-culture with 6 hours matured DCs. We observed enhanced T-cell numbers in short-term compared to long-term co-cultures (OT-I CD8: p = 0.03; OT-II CD4: p = 0.0005; table 3; figure 3C). Similarly, when co-culturing 6 hours LPS/IFN-γ stimulated human DCs with total CD3^+^ human T-cells, proliferation of CD4^+^ as well as CD8^+^ T-cells was enhanced after short-term contact compared to continuous long-term contact conditions (CD4: p = 0.0003; CD8: p = 0.0001; table 3).

**Table 3:** Total cell count of proliferated T-cells after short-term or long-term contact with differently stimulated DCs.

DC maturation		T-cell subset	DC/T-cell contact	Median±SEM (×10^3^)	n	P
LPS/ IFN-γ	6 h	CD4 enriched	short-term	18.0±3.3	8	<0.0001
			long-term	0.7±0.2	8	
	48 h		short-term	12.2±1.7	6	<0.0001
			long-term	0.7±0.2	6	
LPS	6 h		short-term	2.9±0.5	9	<0.0001
			long-term	9.0±0.6	9	
	48 h		short-term	2.2±0.3	6	<0.0001
			long-term	25.7±1.9	6	
Mediator cocktail	6 h		short-term	2.4±1.2	3	0.03
			long-term	8.9±1.6	3	
	48 h		short-term	12.6±2.0	3	0.03
			long-term	22.9±2.3	3	
LPS/ IFN-γ	6 h	CD4 (CD3 enriched)*	short-term	21.5±2.5	11	0.0003
			long-term	4.3±1.0	11	
		CD8 (CD3 enriched)*	short-term	3.8±0.5	11	0.0001
			long-term	1.0±0.3	11	
LPS/ IFN-γ (murine)	6 h	OT II	short-term	22.8±3.5	12	0.0002
			long-term	6.1±1.4	12	
		OT II (mix)**	short-term	3.6±0.2	5	0.0005
			long-term	1.8±0.1	5	
		OT I (mix)**	short-term	3.2±0.7	5	0.03
			long-term	2.0±0.3	5	

* Indicates total CD3 enriched T-cells subjected to short- or long-term contact with DCs; total cell count was assessed by gating on proliferated CD4^+^ or CD8^+^ positive T-cells during analysis.

** Indicates mixture of OT II and OT I T-cells (ratio 4∶1) subjected to short- or long-term contact with DCs; the total cell count was assessed by gating on CD4^+^ (OT II) or CD8^+^ (OT I) positive T-cells during analysis.

### CD40 Ligation as an Early Event in DC/T-cell Interactions Modulated T-cell Proliferative Capacity when Contacted with Pro-inflammatory DCs

**Figure 4:**
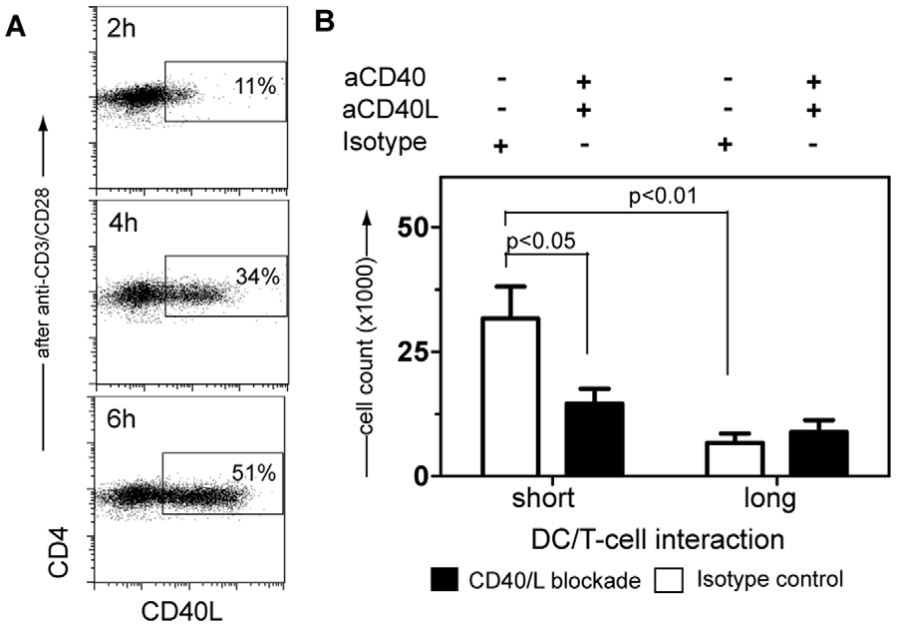
Blockade of CD40/CD40L signaling in short- versus long-term DC/T-cell Co-cultures. (A) CD40L expression on CD4^+^ T-cells activated after 2, 4 and 6 hours of CD3/CD28 cross-linking is shown. One representative out of 4 experiments is depicted. (B) Short- or long-term co-culture of CD3^+^ T-cells and DCs, activated with LPS/IFN-γ for 6 hours, in the presence of blocking CD40 and CD40L monoclonal antibodies (black bars) or isotype control immunoglobulins (white bars) is shown. Median±SEM of 7 to 12 experiments is depicted. DCs from Donors A, B and D were used in experiments (A) and (B).

To better understand the critical mechanisms that cause enhanced T-cell proliferation under short-term co-culture conditions, we investigated the induction of CD40L expression on T-cells that may modulate the DCs’ function by interaction with CD40. On T-cells activated by use of α-CD3/α-CD28 monoclonal antibodies, CD40L expression reached maximum levels already after 6 hours (figure 4A); also we detected up-regulation of CD40 on DCs at early time points after stimulation (figure 1A). This suggests that engagement of CD40 with CD40L may take place during the 6 hours of short-term DC/T-cell contact and modulate the DC maturation signal, which in turn affects T-cell function.

Blockade of CD40/CD40L molecules (figure S3) reduced the proliferation of CD4^+^ T-cells by 51% in short-term co-cultures. In contrast, the overall reduced proliferation during long-term DC/T-cell co-cultures was not affected by CD40/CD40L blockade (figure 4B). We hypothesize that CD40/CD40L signaling in 6 hours pre-matured DCs re-enforces the DC’s early pro-inflammatory mode of action, which is critical for Th1 polarization and enhanced T-cell proliferation. Initiation of a DC/T-cell co-culture after 48 hours of DC pre-maturation also results in T-cells expressing CD40L. However, CD40L engagement on 48 hours pre-matured DCs reinforces their anti-inflammatory mode of action, which primes FoxP3 expressing Tregs.

### Delayed IL-10 Expression Overlaps with IL-12 within the Same DC

**Figure 5:**
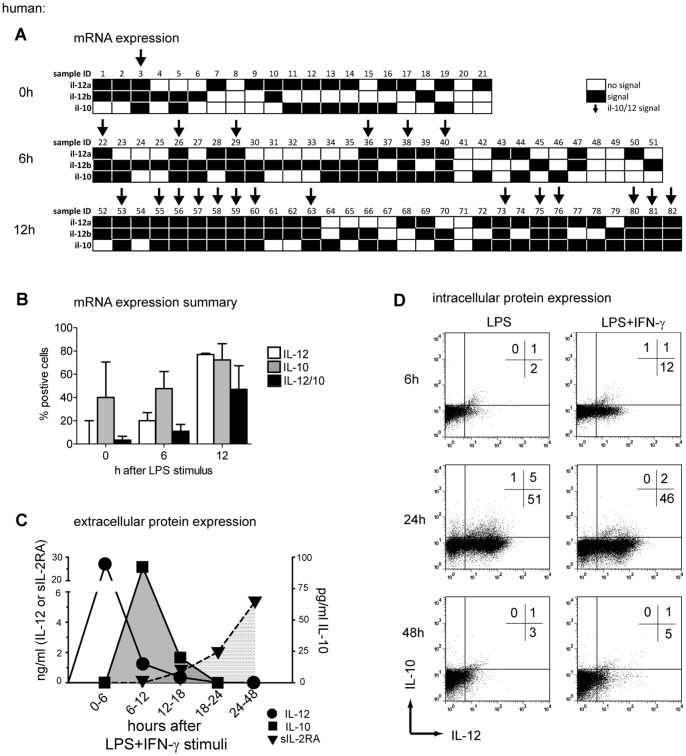
Production of IL-12 and IL-10 on a single-cell level in DCs. (A) Real-Time PCR was used to assess IL-12p35, IL-12p40 and IL-10 mRNA on single cell levels before and after 6 or 12 hours exposure to LPS. All b2m positive sorted single cells were further analyzed for IL-12p35, IL12-p40 and IL-10. Every single cell is depicted (sample ID) for signal (black), no signal (white) and mRNA signal of all three targets (arrow). (B) The summary of 3 independent experiments is depicted as median±SEM. Bars represent the percentage of IL-10 (grey bars), IL-12 (white bars, p35/p40 double positive signals) or IL-10/IL-12 double producing cells (black bars). (C) The concentration of freshly secreted IL-12 (circles), IL-10 (squares), and soluble IL-2RA (triangles) from LPS/IFN-γ activated human DCs is shown in 6 hours intervals after LPS/IFN-γ maturation. At each time point the entire medium was exchanged in order to assess only freshly produced cytokines during the respective time intervals. One representative out of 3 experiments is shown. (D) Intracellular staining of IL-10 and IL-12 protein before and after 6, 24, and 48 hours of exposure to LPS or LPS/IFN-γ. Quadrants are adjusted to the fluorescence of antibody-labeled immature DCs. One representative experiment of 2 is shown. DCs from Donor A and F were used in experiment (A) to (D).

The sequential secretion of cytokines raised the question of whether they were secreted from the same DC undergoing continuous differentiation; or from distinct DC subpopulations, each of which is characterized by the secretion of different cytokines. In order to address this question we investigated the sequential expression of IL-12 and IL-10 within the same cell using quantitative RT-PCR of flow-sorted single DCs after LPS stimulation. Only 5% of immature DCs exhibited signs of simultaneous IL-12p35, IL-12p40, and IL-10 mRNA expression (figure 5A & B). Six hours after LPS exposure, induction of both IL-12p35 and IL-12p40 were detected in 22% of all single DCs, whereas 48% of all single DCs expressed IL-10 mRNA; 19% of all cells did not express any cytokine-related mRNA. However, 11% of these cells produced both IL-12 and IL-10 mRNA at this time point. After 12 hours of LPS activation, 58% of all single DCs expressed IL-12p35 and IL-12p40, and 72% expressed IL-10 mRNA; 47% of single DCs expressed IL-12 as well as IL-10 mRNA 12 hours after activation. The IL-2 receptor α-chain (CD25) may not only be detected as a DC membrane molecule [26], but was also found in the culture supernatant as sIL-2RA. It became detectable 18 hours after the exposure of DCs to LPS/IFN-γ and reached its plateau expression at 48 hours (figure 5C).

To determine the release of IL-10 or IL-12 protein, immature DCs were stimulated with LPS or LPS/IFN-γ and analyzed for intracellular IL-10 and IL-12 expression using flow cytometry (figure 5D). DCs stimulated for 48 hours with LPS alone released more IL-10 into the culture supernatant as compared to LPS/IFN-γ secretion (figure 5C, p = 0.006). We therefore used LPS in comparison to LPS/IFN-γ to induce maximum IL-10 release thus enhancing the sensitivity of our assays. Intracellular accumulation of IL-12 was first detectable 6 hours after initiation of maturation with LPS (2%) and LPS/IFN-γ (12%). At 24 hours, 51% of DCs after LPS and 48% of DCs after LPS/IFN-γ maturation were IL-12 single positive. At 24 hours, IL-10 became detectable in 6% of DCs after LPS and in 2% of DCs after LPS/IFN-γ exposure. At the same time, 5% of LPS and 2% of LPS/IFN-γ matured DCs were double positive for IL-12 and IL-10.

Together, this suggests that both cytokines are produced in parallel within the same DC. At 48 hours, only background levels of IL-12 and IL-10 were detected. The expression pattern of intracellular IL-12 and IL-10, induced with LPS or LPS/IFN-γ, correlates with cytokine release into the culture supernatant (figure 5C & D). We also detected IL-10 mRNA at time points when no IL-10 protein was measurable, like in un-stimulated DCs or 6 hours after an LPS stimulus (figure 5). As reported earlier this effect might be due to the rigorous regulation of IL-10 on a post-transcriptional level [18].

## Discussion

We describe the induction of a dynamically changing differentiation program in DCs. During the first 24 hours after exposure to LPS/IFN-γ, DCs assume a pro-inflammatory Th1 polarizing mode. At 24 hours, DCs switch into an anti-inflammatory mode favoring Treg priming. We found that T-cell priming only requires a few hours of contact with 6 hours LPS/IFN-γ matured DCs. Thus, the transient immune stimulatory time window of pro-inflammatory DC activity suffices for T-cell priming and polarization. This notion is supported by the observation of a sequential expression of molecules with opposing functions in DCs, like the strongly pro-inflammatory cytokine IL-12, as well as the anti-inflammatory molecules IL-10, p-STAT3 and sIL-2RA. Both, human allogeneic T-cells as well as syngeneic murine ovalbumin specific T-cell receptor transgenic OT-I/II cells proliferated several-fold stronger when cultured alone after a few hours of DC-contact, as opposed to a continuous DC/T-cell co-culture. The T-cell cytokine secretion profile after short-term contact with DCs in their pro-inflammatory mode resulted in a dominant type 1 T-helper cell polarization and reduced priming of Tregs. These experiments strongly suggest that DCs not only branch out into subtypes [2] but also execute a time kinetic differentiation program upon encountering the microbial stress signal LPS.

It had already been shown that the kinetics of activation influences the capacity of DCs to induce different types of T-cell responses [14]. Experiments performed by us [15], [17] and others [4], [16] strongly hinted at the need for DC/T-cell interaction during the immune stimulatory phase in the presence of IL-12 secretion, which is limited to a short time span after activation [14] and can be boosted by addition of IFN-γ to the maturation cocktail [27]. We demonstrate that only 6 hours contact between T-cells and IL-12 producing DCs is enough to trigger potent T-cell activation with a Th1 polarization profile. In contrast, continuous DC/T-cell contact, like it is done in essentially all conventional in vitro assays, triggered a differentiation profile dominated by Tregs. Our in vitro observations are in line with in vivo studies of DC/T-cell interactions in functionally intact lymph nodes using two-photon microscopy [28]. It was also shown that transient DC/T-cell interaction, that lasted between 1 to 30 minutes, lead to T-cell activation and proliferation [29] and were mainly detected within the first 8 hours of interaction [30]. In vitro, the culture vessel artificially forces prolonged DC/T-cell contact after the DC’s switch from the pro- into the anti-inflammatory mode of action. This results in the exposure of T-cells to signals from DCs after their phenotypic switch into the anti-inflammatory mode. Under physiologic conditions, primed T-cells will leave the lymph node to engage in their search and destroy mission thus dodging the anti-inflammatory signals from the maturing DCs.

Several lines of evidence support our notion of the DCs’ phenotypic switch from an immune stimulatory pro-inflammatory into an immune suppressive anti-inflammatory mode. First, studies of the enzyme IDO [22] revealed its immune suppressive function in DCs. Co-cultivating T-cells with IDO^+^ DCs lead to suppression of effector T-cell proliferation and priming of Tregs [23]. Second, high surface expression of the IL-2 receptor and secretion of the IL-2RA molecule was identified in DCs late after LPS exposure [26], [31]. Secretion of the soluble IL2-RA was described recently to induce Treg cell priming [32], [33], [34]. IL-10 is another molecule released from DCs that is strongly linked to immune suppression [18]. In DCs, IL-10 acts as a feedback regulator of Th1 responses [35], [36] and causes autocrine phosphorylation of the anti-inflammatory signal transducer molecule STAT3 [37]. Besides the detection of a switch from IL-12 to IL-10 secretion only a few hours after maturation, we found STAT3 phosphorylation and expression of sIL-2RA in DCs one day after exposure to LPS. Together these observations serve as additional evidence to support our hypothesis of a time dependent functional change of DCs following an encounter with a microbial danger signal.

The implication of earlier murine studies is that DC differentiation branches out into subsets with each DC subset representing one distinct functional phenotype [2]. Thus, an alternative interpretation of our observations is that the population of human monocyte and murine stem cell derived DCs branches into two distinct DC subtypes: one subtype would proceed under direction of TLR signaling to become an IL-12 secreting immune stimulatory DC, whereas another subtype under identical conditions would differentiate into an IL-10 secreting immune suppressive phenotype. To address this question we chose two different detection systems based on single cell analysis: First, we measured the mRNA level of IL-12 and IL-10 in flow-sorted single cells. This enabled us to demonstrate that IL-12 and IL-10 mRNA was detectable in the same DC at around 12 hours after encountering LPS. Obviously this overlap of the expression of both IL-12 and IL-10 mRNA is limited to a brief time period while the DC switches from the pro-inflammatory immune stimulatory into the anti-inflammatory immune suppressive mode. Furthermore, IL-12 and IL-10 protein passing through the Golgi apparatus was measured by intracellular flow cytometry.

As third method for measuring cytokine secretion we used ELISA. We found considerable amounts of both, IL-12 and IL-10 in the supernatant of DC cultures. Especially IL-10 secretion appeared high in light of the relatively low number of IL-10 positive DC in the intracellular staining. The reason for this might be that ELISA measures accumulated cytokines in the culture supernatant, whereas the intracellular staining is more like a snap shot of the cytokines in the cell. Another explanation for the low levels of intracellular accumulated IL-10 protein might be that the auto-regulative positive feedback mechanism of IL-10 synthesis is disrupted by blocking its secretion and thus the binding to the IL-10 receptor [37].

These experiments confirmed that an individual DC is capable of producing both, IL-10 as well as IL-12 in a consecutive and briefly overlapping manner. Obviously, time dependent DC-mediated immune regulation doesn’t preclude the existence of functionally varying DC subsets. We suggest therefore that DC subsets and time-dependent DC differentiation represent two layers of immune regulation that are superimposed on each other, allowing the fine-tuning of immune responses to various environmental threats.

Many immunologists see the danger model [6] as a helpful framework for understanding immune regulation, especially in the case of anti-tumor immunity. DCs continuously take up, process, and present material mainly from apoptotic cells. This holds true for DCs situated in the tumor tissue as well. As opposed to microorganisms, however, tumor cells don’t deliver danger signals to DCs and therefore tumor antigens are presented to T-cells in a tolerance inducing fashion. Hence, the immune system itself provides a useful loophole allowing tumor cells to evade immune surveillance. Adding a TLR derived danger signal to the equation tips the balance towards immune stimulation. Consequently, while earlier DC cancer vaccine designs advocated maturation of DCs with a mediator cocktail comprised of TNF-α, PG-E2, IL-1β, and IL-6 [9], more recent strategies for DC based cancer immune therapy utilize TLR agonists in order to trigger IL-12 secretion from the DCs causing differentiation of Th1 and CTL priming human DCs: polyI:C engagement of TLR3 [38], [39], [40]; LPS binding to TLR4 [5], [25], [41], [42], [43], [44]; or synthetic TLR7/8 agonists such as R848 [45], [46]. Our data also confirm earlier observations [47] that a combination of a TLR ligand with IFN-γ results in the most powerful IL-12 release from human DCs. However, in most of these DC cancer vaccine strategies, the DCs were inoculated after at least 24 hours of in vitro exposure to LPS/IFN-γ. In contrast, in the design of our clinical DC cancer immune therapy trials we elected to inoculate the DCs after only 6 hours of in vitro maturation in order to take advantage of the immune stimulatory time window characterized by IL-12 release [5], [25].

Using DCs in cancer immune therapy effectively necessitates an understanding of their pharmacodynamic and pharmacokinetic features, similar to the knowledge required when using traditional drugs. Using a living DC in cancer immune therapy, we find in addition to the interactions between cellular medicine and diseased organism the DC’s intrinsic differentiation program. The utilization of DCs in cancer immune therapy requires that the specific features of human DCs are well understood in order to optimally exploit their therapeutic capacity. Therefore it is of critical importance to continue this line of research in order to improve the use of DCs as medicines in cancer immune therapy and other ailments mediated by the immune system.

In summary, we observed that separation of T-cells from DCs after a 6 hours interaction and re-cultivating the T-cells alone resulted in a more potent proliferative capacity compared to a continuous DC/T-cell co-culture. This was accompanied by a type 1 cytokine secretion profile favoring Th1 polarization while disfavoring Treg priming. We suggest that microbial danger signals such as LPS in the presence of IFN-γ initiate a dynamically changing differentiation program that drives the DC from a tolerance-maintaining mode to assume a potently pro-inflammatory immune stimulatory phenotype. After about one day, the DC switches into an anti-inflammatory immune suppressive mode that has the capacity to down-modulate the activity of T-cells. Such time dependent DC-mediated immune regulation doesn’t rule out the existence of DC subsets with specialized functions. We conclude that an immune response is regulated not only by DC subtypes with distinct functions but is also fine-tuned by a time kinetic differentiation program that is initiated by TLR signaling. Our observations will need to be taken into consideration in designing DC based cancer immune therapy protocols.

## Methods

### Ethics Statement

The use of peripheral blood from healthy adult volunteers was approved by the institutional review board of the St. Anna Children’s Cancer Research Institute and conducted according to the Declaration of Helsinki. Peripheral blood mononuclear cells were collected using leukocyte apheresis, to which all volunteers gave written informed consent.

No in vivo mouse experiments were performed. Mice were sacrificed by cervical dislocation and spleens as well as lymph nodes were harvested from the corpses. Collection of organs was registered at the institutional review board of the Medical University of Vienna.

### Mice

C57BL/6, OT-II and OT-I transgenic mice, purchased from the Research Institute for Laboratory Animal Breeding, University of Vienna (Himberg, Austria) were housed at the animal care unit of the Department of Pharmacology, Medical University of Vienna, Austria.

### Murine Dendritic Cells

Dendritic cells were generated as previously described [17]. Briefly, murine bone marrow cells were harvested from femur and tibia and re-suspended in IMDM medium (GIBCO/Invitrogen) supplemented with 10% fetal calf serum (PAA) 1 mM Sodium Pyrovate, non-essential amino acids, Penicillin/Streptomycin (GIBCO) and 0.0002% b-Mercaptoethanol (Sigma). Bone-marrow cells were plated at a density of 1×10^6^ cells/ml/cm^2^ with 5 ng/ml recombinant murine IL-4 and 3 ng/ml recombinant murine GM-CSF (Peprotech). Loading of DCs with 1 µg/ml SIINFEKL and/or Ovalbumin (Bachem) was initiated 2 hours prior to maturation; the peptide remained in the DC culture during maturation. For maturation 1 µg/ml LPS (E. coli strain O111:B4, Calbiotech) in combination with 0.02 µg/ml IFN-γ (BD Pharmingen) was added for 4 hours.

#### Human dendritic cells

Isolation of human monocytes and peripheral blood leucocytes (PBLs) were performed as described previously [48]. Monocytes were differentiated into DCs following a previously optimized protocol [5], [49], in CellGro DC medium (Cellgenix) supplemented with 1000 U/ml recombinant human GM-CSF and 300 U/ml recombinant human IL-4 (both from Peprotec).

DC maturation was achieved using *(i)* 100–1000 ng/ml LPS (E. coli strain O111:B4, Calbiochem) alone (table S1), or *(ii)* in addition with 50 ng/ml IFN-γ (Imukin, Boehringer Ingelheim), or *(iii)* mediator cocktail, comprised of a combination of 1 µg/ml PGE2, 10 ng/ml IL-1β, 100 U/ml IL-6 (all from Peprotec), 1000 U/ml TNF-α (Sigma). After 6 or 48 hours cells were frozen in 10% DMSO, 40% fetal calf serum and 50% CellGro medium. After recovering DCs from freezing, the amount of viable cells was determined using DAPI; in all subsequent cultures >95% of DCs were alive.

### Human and Murine T-cells

Human allogeneic PBLs and murine OT-I or OT-II splenocytes were enriched by negative depletion using MACS (Miltenyi) for CD3^+^, CD4^+^ or CD8^+^ T-cells and/or labeled with proliferation tracker 5(6)-Carboxyfluorescein diacetate N-succinimidyl ester (CFSE, Sigma) at a final concentration of 7 µM.

### Contact-restricted DC/T-cell Co-cultures

Pre-matured DCs and enriched T-cells were co-cultured at a DC:T-cell ratio of 1∶1 for 6 (human) or 12 hours (murine). After this initial contact-phase the cells were flow sorted using a FACSAria 2 flow cytometer (BD Biosciences). T-cells are separated from DC according to morphology and their CFSE positive signal in order to avoid any manipulation of the cells with monoclonal antibodies. (figure S2). The conditioned supernatant of DC/T-cell co-cultures was saved for further cultivation. The separated T-cells were cultured for 3 (murine) or 6 days (human) in conditioned medium either alone (here termed “short-term” co-culture) at a density of 0.5×10^6^ cells/ml or with DCs at a concentration of 1×10^6^ cells/ml with a final DC:T-cell ratio of 1∶1 (here termed “long-term” co-culture). Murine syngeneic CD4^+^ T-cells, or a mix of CD4^+^ and CD8^+^ T-cells at a ratio of 4∶1 was used (referred to as “mix”) and cells were cultured in murine culture medium. Human allogeneic DC:T-cell co-culture was performed in AIM-V (Invitrogen), supplemented with 2% pooled human AB plasma (Octaplas, Octapharma).

### Western Immunoblots

T-cells were collected after 6 days co-culture, lysed in RIPA and 4×10^4^ cells were subjected to electrophoresis on 10% acrylamid gels and transferred to nitrocellulose membranes (Whatman). Western Blocking reagent (Applied Biosystems) was used for decreasing unspecific binding. Antibodies against human FoxP3 (Biolegend) and GAPDH (Applied Biosystems) were used at a dilution of 1∶250 or 1∶4000, respectively, followed by a peroxidase-conjugated anti-rabbit IgG (Thermo Scientific, 1∶10000). Blots were developed using a chemoluminescent Detection Film (Lumi-Film, Roche) and chemoluminescent kit (West-Femto, Thermo Scientific) according to the manufacturer’s instructions. As a positive control for FoxP3 expression a human FoxP3 transfected cell lysate (Biolegend) was used.

### Real-time Polymerase Chain Reaction (RT-PCR) Analysis

Un-stimulated and 6 or 12 hours LPS matured human DCs were single cell flow sorted using the single-cell modus of FACSAria 2 flow cytometer (BD Biosciences). The sorting procedure was confirmed by light microscopy and by b2-microglobulin (b2m) expression as previously described [50]. For every condition 10 single DCs were lysed separately. Messenger RNA was extracted from single cells using the Single-Cell-to-CT Kit (Ambion) according to the manufacturer’s guidelines. Sequences of primers (Eurogentec) used for IL-12p35 were 5′-CCTGGACCACCTCAGTTTGG-3′ sense, 5′-TGAAGGCATGGGAACATTCC-3′ antisense; IL-12p40∶5′-GATGCCGTTCACAAGCTCAA-3′ sense, 5′- AGTTCTTGGGTGGGTCAGGTT -3′ antisense; IL-10∶5′-CCAGGGCACCCAGTCTGA-3′ sense, 5′-TCGGAGATCTCGAAGCATGTT-3′ antisense; b2m: 5′-TGAGTATGCCTGCCGTGTGA-3′ sense, 5′-TGATGCTGCTTACATGTCTCGAT-3′ antisense. Sequences for probes (Metabion) used for IL-12p35 were 5′-FAM-CAGAAACCTCCCCGTGGCCACTC-TAMRA-3′ sense; IL-12p40∶5′-FAM-CCAGCAGCTTCTTCATCAGGGACATCA-TAMRA-3′ sense; IL-10∶5′-FAM-AGCTGCACCCACTTCCCAGGCAA-TAMRA-3′ sense; b2m: 5′-FAM-CCATGTGACTTTGTCACAGCCCAAGATAGTT-TAMRA-3′ sense. Primers were used at a final concentration of 900 nM each, probes at 250 nM according to the instructions of the kit. Amplifications were conducted on a 7500 Fast Real-Time PCR system cycler (Applied Bioscience) according to the manufacturer’s instructions. All reactions were done in duplicates and finished with a melting curve run to establish the specificity of the PCR. Only single DCs in which the intensity of the b2m signal indicated the presence of one cell per well were further analyzed for IL-12p35, IL-12p40 and/or IL-10 mRNA expression. Melting curves of b2m with high CT values were excluded using median±2*SD test of outliers. Percentage of single cells expressing IL-12p35, IL-12p40 and/or IL-10 was assessed of all b2m positive signals.

### Cytokine Measurement

Supernatants of DCs or T-cells (re-stimulated after the primary culture with anti-CD3 for 24 hours), both cultured at 1×10^6^ cells/ml were analyzed using the Flow Cytomix system (eBiosciences) following the manufacturer’s protocol. Detection of sIL-2RA was assessed using ELISA (eBiosciences). If not stated otherwise, all cytokines were accumulated during the indicated time of the culture. For the detection of freshly secreted cytokine detection (figure 5C), the medium was replaced completely every 6 hours over a total culture period of 48 hours.

### Flow Cytometry Analysis and Sorting

For surface molecule detection cells were labeled with antibodies for 15 min at 4°C. Elutriation products were analyzed as described before [5]. The total cell count was assessed using Trucount tubes (BD Biosciences). T-cell activation and proliferation was measured by CFSE dilution and DAPI (Sigma) was used for life/dead discrimination. FACS acquisition was performed on a FACSCalibur or LSR 2 flow cytometer (BD Biosciences). Further analysis was performed using FlowJo software Version 6.7.1 (Treestar).

For intracellular cytokine detection, we used Golgi Stop (BD biosciences) 6 to 12 hours before fixation with 2% formaldehyde (Polysciences). Permeabilization of fixed cells was performed with 0.1% Triton (Roth). To block unspecific binding MOPC (SIGMA) was added 10 minutes before staining with fluorescent anti-human APC-anti IL-10 (clone JES3-9D7) and FITC-anti IL12p40/70 (eBioscience, clone C8.6). For intracellular detection of p-STAT3 we used methanol permeabilisation and stained with Alexa Fluor® 647-antiSTAT3 (clone pY705) (BD Biosciences).

To divide DCs and T-cells after the initial co-culture, cell-aggregates were dissolved by AccuMAX (PAA) and flow-sorted using a FACSAria 2 flow cytometer (BD Biosciences) according to their morphology into DC and T-cell subsets. In addition, we used CFSE positivity to discriminate between DCs and T-cells in the human system and excluded cell-aggregates by sorting only FSC-width negative cells.

### Fluorochrome Labeled Monoclonal Antibodies

The following anti-human mABs were used according to their datasheet for surface molecule detection of DCs or T-cells: FITC-anti HLA-DP/DQ/DR (clone CE3/43), FITC-anti CD1a (clone HI149), PE-anti HLA-ABC (clone W6/32) (Dako), APC-anti CD83 (clone HB15e), APC-anti CD86 (clone 2331; FUN-1), PerCP-anti CD45 (clone CD1), PE-anti CD40L (clone TRAP1), FITC-anti CD40 (clone 5C3), PE-TR-anti CD3 (custom conjugate, clone UCHT1), PerCP-anti CD4 (clone SK3), APC-Cy™7-anti CD8 (clone SK1), PE-Cy™7-anti CD56 (clone NCAM162) (BD Pharmingen), PE-anti CD80 (clone MAB104) (Beckman Coulter) and Alexa Fluor® 647-anti CD25 (clone MCA2127A647) (Serotec).

The following anti-mouse mABs were used according to their datasheet for surface molecule detection of DCs or T-cells: APC-Cy™7-anti CD11b (clone M1/70), PE-Cy™7-anti CD11c (clone HL3), APC-anti CD3e (clone 145-2C11), APC-Cy™7-anti CD8a (clone 53-6.7), FITC-anti CD4 (clone H129.19) FITC-anti H-2D[b] (clone KH95), PE-anti I-A[b] (clone AF6-120.1) (BD Pharmingen), PerCP-Cy5.5-anti CD80 (clone 16-10A1) and Alexa Fluor® 700-anti CD86 (clone PO3) (Biolegend).

### CD40/CD40L Blockade

CD40/CD40L signalling was blocked using purified NA/LE anti-CD40 (clone 5C3) and/or CD40L (clone TRAP1) monoclonal antibodies (BD Pharmingen). In preliminary experiments we established the concentration of anti-CD40/anti-CD40L for blocking CD40/CD40L-mediated signalling [51]. We used a CD40L mediated maturation signal delivered to DC from CD40L transgenic SJ-NB-7 cells. The DC/SJ-NB-7-CD40L co-cultures were supplemented with increasing concentrations of CD40 and/or CD40L monoclonal antibodies. We found the optimal concentration of blocking CD40/CD40L signalling was 30 µg/ml of antibody. A combination of both, CD40 and CD40L monoclonal antibodies was found to be capable of entirely preventing the secretion of IL-12 from DC co-cultivated with SJ-NB-7-CD40L (figure S3). Thus, we used a combination of CD40 and CD40L monoclonal antibodies in our experiments aimed at elucidating the impact of CD40/CD40L signalling in short- and long-term DC/T-cell co-cultures (Figure 4).

### Statistical Analysis

Two tailed p-values were calculated using the non-parametric Mann-Whitney test to determine statistical significance using GraphPad Prism version 5.02 for Windows (GraphPad Software). All data are given as median±SEM.

## Supporting Information

Figure S1 **STAT3 expression in DC.** (A) Comparison of STAT3 phosphorylation levels in human DCs after exposure to LPS (black line), LPS/IFN-γ (dotted line), IFN-γ alone (black hairline), or un-stimulated DCs (filled gray histogram). One representative experiment out of 2 is shown. (B) STAT3 phosphorylation was measured in un-stimulated human DCs (filled gray histogram) and at the indicated time points after DC activation with LPS (black line). One experiment out of 2 is shown. DCs from Donor F were used in experiment (A) to (B).
(TIFF)

Figure S2 **DC/T-cell co-culture sorting strategy.** Human DCs and T-cells (TCs) were co-cultured for 6 hours before flow sorting based on their mophological differences in size and granulosity and the CFSE positivity of T-cells. Unsorted DC/T-cell co-cultures (A), T-cell (B) and DC (C) fraction after flow sorting are shown as one representative out of 13 independent experiments. Absolute cell count of viable, DAPI negative cells was assessed using BD Trucount beads (indicated as “beads”).
(TIFF)

Figure S3 **Blocking CD40/CD40L signaling.** Immature DC were exposed to LPS/IFN-γ or co-cultivated with a cell line engineered to express CD40L molecules (SJ-NB-7/CD40L). For blocking CD40/CD40L-mediated maturation signals in DCs the co-cultures we supplemented with reactive NA/LE anti-human CD40, CD40L, or both. The mean fluorescence intensity (MFI) of the indicated DC membrane molecules was analyzed using flow cytometry. Shown are median±SD of three independent experiments. The relatively high expression density of some of the maturation markers on immature DCs is a result of the serum free culture conditions, which cause cellular stress and somewhat enhanced cell death representing a damage-associated molecular pattern resulting in elevated baseline expression levels of the DC’s membrane molecules, but no IL-12 secretion.
(TIFF)

Table S1 **Concentration of LPS used for maturation of DC from different donors.**
(DOCX)
