# Supplementary figures and images for: Toll-Like Receptor 4 Engagement Drives Differentiation of Human and Murine Dendritic Cells from a Pro- into an Anti-Inflammatory Mode
Source: PLoS One. 2013 Feb 11;8(2):e54879. doi: 10.1371/journal.pone.0054879 (PMC3569454; doi:10.1371/journal.pone.0054879)

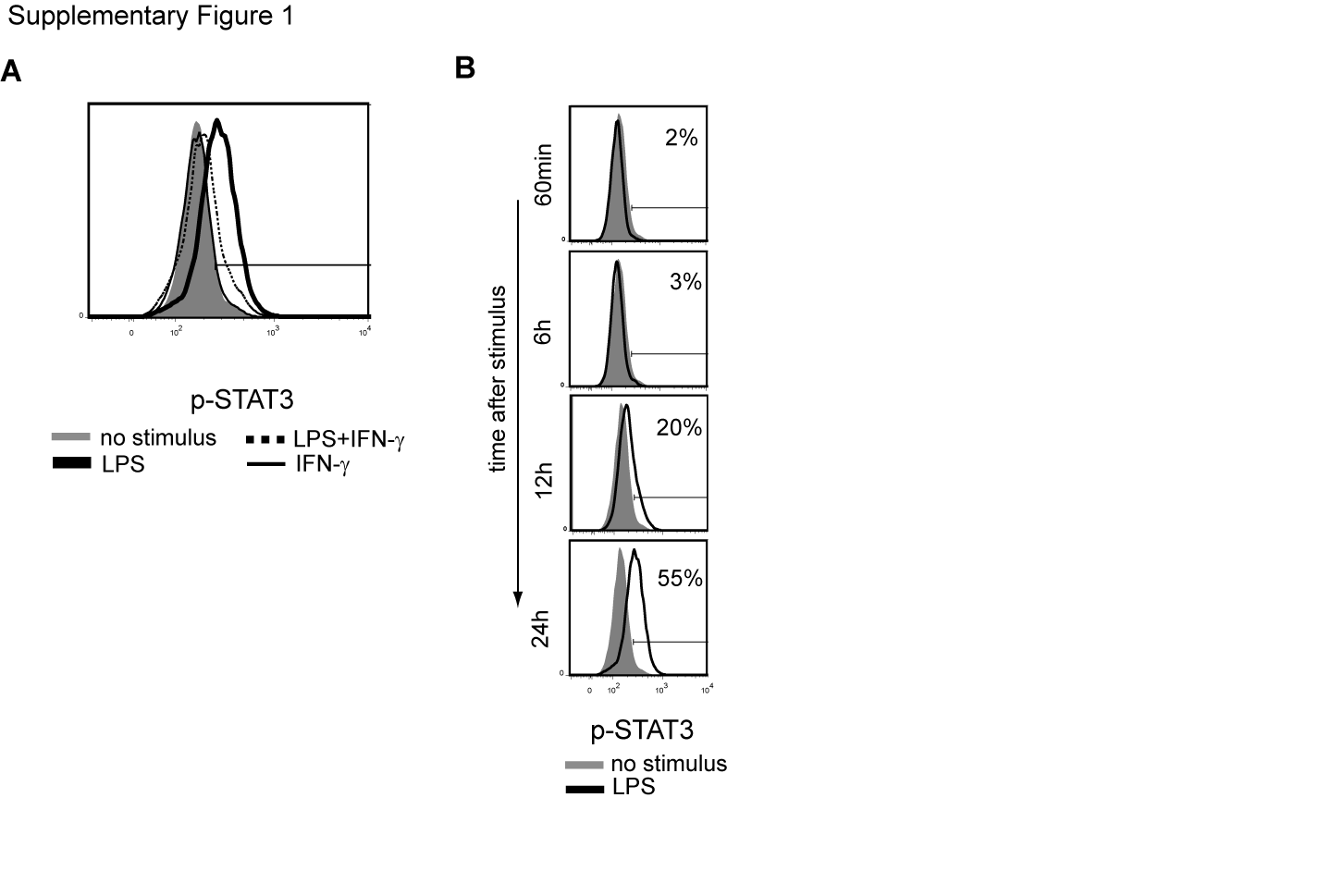

Supplement: Figure S1 — STAT3 expression in DC. (A) Comparison of STAT3 phosphorylation levels in human DCs after exposure to LPS (black line), LPS/IFN-γ (dotted line), IFN-γ alone (black hairline), or un-stimulated DCs (filled gray histogram). One representative experiment out of 2 is shown. (B) STAT3 phosphorylation was measured in un-stimulated human DCs (filled gray histogram) and at the indicated time points after DC activation with LPS (black line). One experiment out of 2 is shown. DCs from Donor F were used in experiment (A) to (B). (TIFF) [file pone.0054879.s001.tiff]

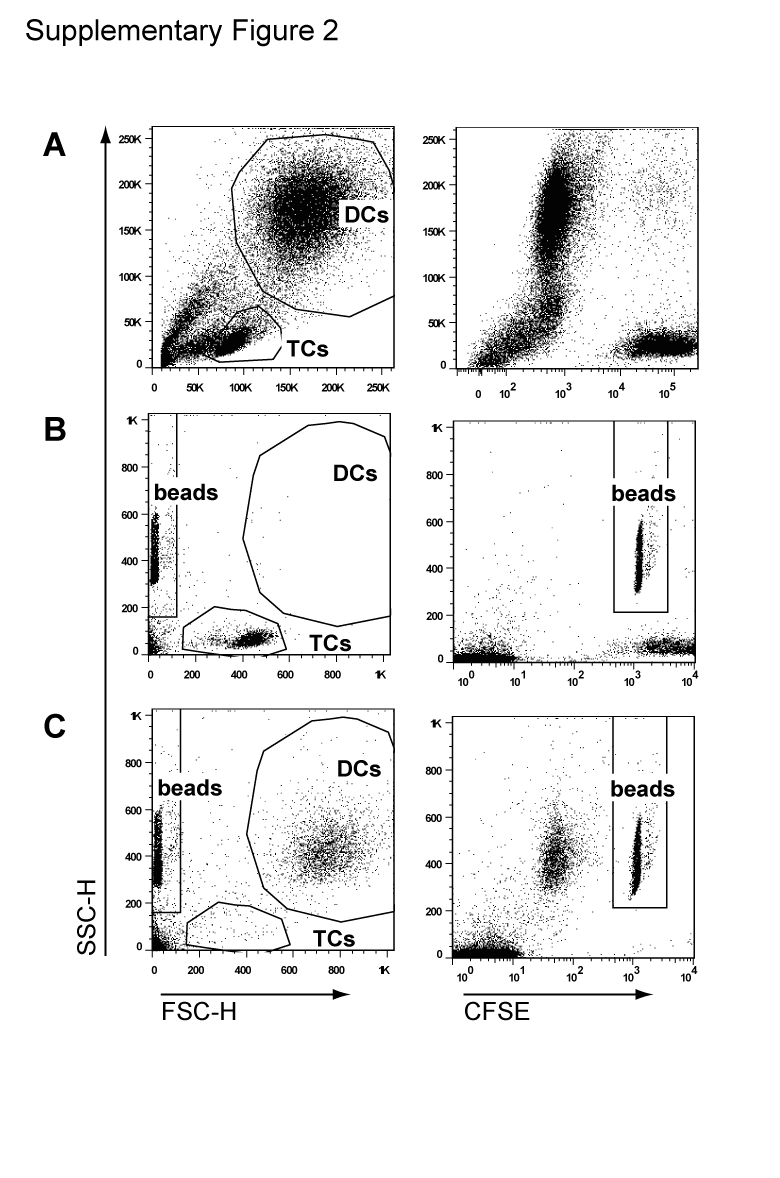

Supplement: Figure S2 — DC/T-cell co-culture sorting strategy. Human DCs and T-cells (TCs) were co-cultured for 6 hours before flow sorting based on their mophological differences in size and granulosity and the CFSE positivity of T-cells. Unsorted DC/T-cell co-cultures (A), T-cell (B) and DC (C) fraction after flow sorting are shown as one representative out of 13 independent experiments. Absolute cell count of viable, DAPI negative cells was assessed using BD Trucount beads (indicated as “beads”). (TIFF) [file pone.0054879.s002.tiff]

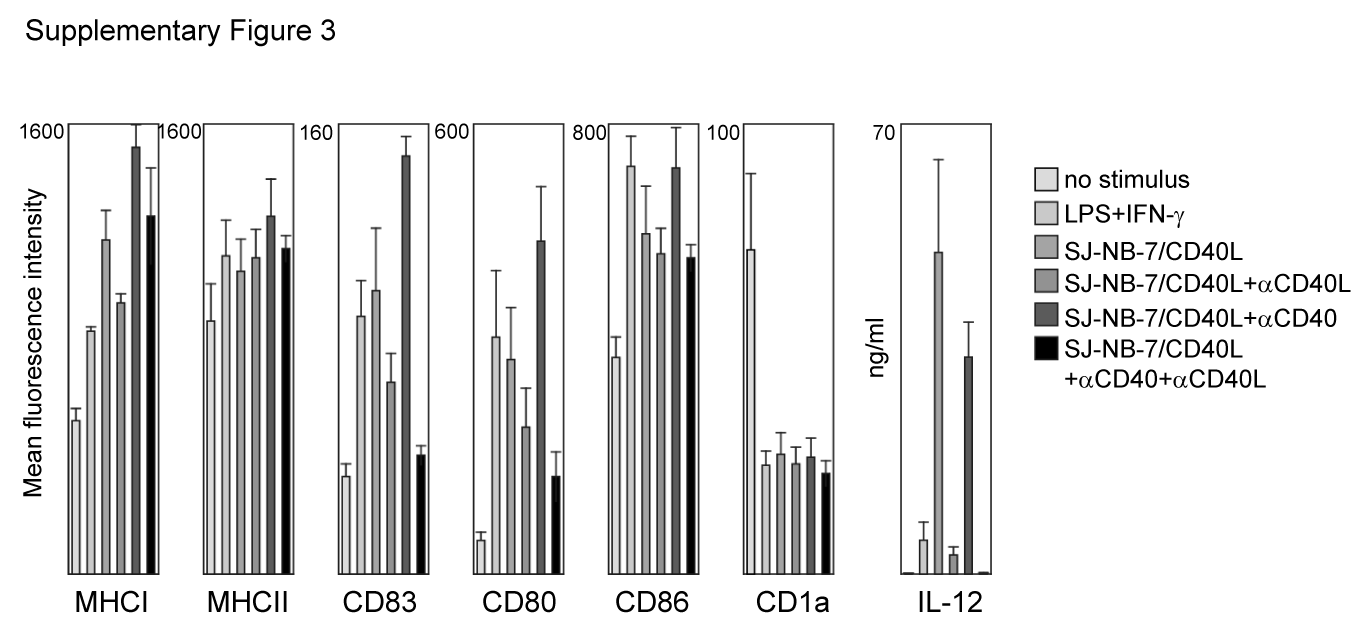

Supplement: Figure S3 — Blocking CD40/CD40L signaling. Immature DC were exposed to LPS/IFN-γ or co-cultivated with a cell line engineered to express CD40L molecules (SJ-NB-7/CD40L). For blocking CD40/CD40L-mediated maturation signals in DCs the co-cultures we supplemented with reactive NA/LE anti-human CD40, CD40L, or both. The mean fluorescence intensity (MFI) of the indicated DC membrane molecules was analyzed using flow cytometry. Shown are median±SD of three independent experiments. The relatively high expression density of some of the maturation markers on immature DCs is a result of the serum free culture conditions, which cause cellular stress and somewhat enhanced cell death representing a damage-associated molecular pattern resulting in elevated baseline expression levels of the DC’s membrane molecules, but no IL-12 secretion. (TIFF) [file pone.0054879.s003.tiff]
