# Supplementary material for: Toll-Like Receptor 4 Engagement Drives Differentiation of Human and Murine Dendritic Cells from a Pro- into an Anti-Inflammatory Mode
Source: PLoS One. 2013 Feb 11;8(2):e54879. doi: 10.1371/journal.pone.0054879 (PMC3569454; doi:10.1371/journal.pone.0054879)
Supplement: Table S1 — Concentration of LPS used for maturation of DC from different donors. (DOCX) [file pone.0054879.s004.docx]

## ***Table S1: Concentration of LPS used for maturation of DC from different donors.***

|  |  | fold increase (ng/ml or MFI) of LPS + IFN-γ stimulated vs. not stimulated DCs | | | |  |
| --- | --- | --- | --- | --- | --- | --- |
| Donor | LPS (ng/ml) | IL-10 | IL-12 | CD80 | CD86 | used in following experiments : |
| A | 1500 | 1,0 | 37,3 | 7,5 | 26,4 | figure 1, 3, 4, 5 |
| B | 700 | 8,8 | 3,6 | 3,1 | 3,9 | figure 1, 3, 4 |
| C | 1000 | 3,8 | 6,0 | 8,2 | 9,2 | figure 1, 2 |
| D | 1000 | 7,6 | 2,4 | 2,2 | 3,0 | figure 1, 3, 4 |
| E | 300 | 0,2 | 212,0 | 7,4 | 11,3 | figure 2 |
| F | 300 | 1,2 | 361,3 | 9,6 | 11,4 | figure 2, 5, suppl. figure 1 |
